# Association of the rs3039851 Insertion/Deletion in the Gene *PPP3R1*, Which Encodes the Regulatory Calcineurin Subunit B Type 1, with Left Ventricular Mass in Polish Full-Term Newborns

**DOI:** 10.3390/biomedicines11051415

**Published:** 2023-05-10

**Authors:** Iwona Gorący, Beata Łoniewska, Klaudyna Lewandowska, Agnieszka Boroń, Małgorzata Grzegorczyk, Robert Nowak, Jeremy Simon C. Clark, Andrzej Ciechanowicz

**Affiliations:** 1 Department of Clinical and Molecular Biochemistry, Pomeranian Medical University, 70-111 Szczecin, Poland; iwona.goracy@pum.edu.pl (I.G.); klaudyna.lewandowska@pum.edu.pl (K.L.); agnieszka.boron@pum.edu.pl (A.B.); malgorzata.grzegorczyk@pum.edu.pl (M.G.); jeremy.clark@pum.edu.pl (J.S.C.C.); 2 Department of Neonatal Diseases, Pomeranian Medical University, 70-111 Szczecin, Poland; beata.loniewska@pum.edu.pl; 3 Institute of Physical Culture Sciences, University of Szczecin, 71-650 Szczecin, Poland; robert.nowak@usz.edu.pl; 4 Department of Pathology, Pomeranian Medical University, 70-111 Szczecin, Poland

**Keywords:** calcineurin, gene polymorphism, left ventricular mass, neonates

## Abstract

Background: The five base-pair (bp) insertion/deletion (rs3039851) polymorphism in the *PPP3R1* gene, which encodes calcineurin subunit B type 1, has been found to be associated with left ventricular hypertrophy (LVH) in hypertensive patients and in athletes. The aim of this study is to analyze the possible association between *PPP3R1*:rs3039851 polymorphism and left ventricular mass (LVM) in full-term healthy newborns. Methods: The study group consisted of 162 consecutive, full-term, healthy newborns. Two-dimensional M-mode echocardiography was used to assess LVM. The *PPP3R1*:rs3039851 polymorphism was identified by PCR-RFLP in genomic DNA extracted from cord blood leukocytes. Results: No significant differences were found between newborns homozygous for the reference allele (5I/5I, n = 135) and newborns carrying at least one 5D allele (n = 27) for LVM standardized for body mass, body length or body surface area (LVM/BM, LVM/BL or LVM/BSA, respectively). However, the frequency of *PPP3R1*:rs3039851 genotypes with a 5D allele (5I/5D + 5D/5D) among newborns with the largest LVM/BM or LVM/BSA (upper tertile) was statistically significantly higher compared with the prevalence in individuals with the lowest values of both indices (lower tertile). Conclusions: Our results suggest that the *PPP3R1*:rs3039851 polymorphism may contribute to subtle variation in left ventricular mass at birth.

## 1. Introduction

Left ventricular hypertrophy (LVH) and increased left ventricular mass (LVM) are strong risk factors for cardiovascular disease and morbidity [1]. Cardiac hypertrophy is characterized by increased cell size, cardiac remodeling of myofilaments and increased expression of fetal genes [2]. Using twin [3,4] and population studies [5,6], genetic factors are estimated to be responsible for between 30% and 70% of cardiac mass variability [7]. Calcium (Ca^2+^) is the most important messenger in cardiac muscle and plays a central role in regulating contractility, gene expression, apoptosis and hypertrophy. Ca^2+^ transient movements regulate the transcription and gene expression that characterize the hypertrophic response of cardiomyocytes [8].

Calcineurin, a heterodimer composed of a 60 kDa catalytic subunit A (Serine/threonine-protein phosphatase; UNIPROT: A0A0S2Z4C6, P16298 or P48454; from genes *PPP3CA*, *PPP3CB* or *PPP3CC,* respectively) and a 19 kDa regulatory subunit B (Calcineurin subunit B; UNIPROT: P63098 or Q96LZ3; from genes *PPP3R1* or *PPP3R2*), is a calcium- and calmodulin-dependent serine/threonine phosphatase that becomes activated in the presence of increased intracellular Ca^2+^ levels [9]. The exact ways in which changes in calcineurin subunit B could be critical for development of left ventricular mass in full-term healthy newborns have not been fully defined. It is widely recognized that changes in calcineurin activity in the heart contribute to the determination of cardiovascular phenotypes including pathological hypertrophic remodeling and the progression to failure (Table 1).

Of the two genes which encode regulatory B subunits, only the *PPP3R1* gene, which encodes for subunit B type 1, is expressed in the heart (i.e., *PPP3R2* is not expressed in the heart) [20]. In 2005, Tang et al. identified the polymorphism rs3039851 as an insertion (allele 5I) or deletion (allele 5D) of 5 base-pairs (TTAAA) at sequence positions from −1059 to −1063 relative to the transcription start site of the *PPP3R1* gene transcript available at the time (Genbank accession no. NT022184-12; now replaced) [21]. Tang et al. found no association of *PPP3R1*:rs3039851 with traditionally defined LVH in 368 severely hypertensive participants (including 197 African-Americans) in the Hypertension Genetic Epidemiology Network (HyperGEN) study. However, the *PPP3R1* 5D allele in HyperGEN patients was associated with “inappropriately-high” LVM, defined as greater than 128% (which is the upper 90% confidence limit) of the quotient of actual and expected LVM, and this association was independent of age, sex, body mass index, heart rate, prevalent coronary heart disease and/or diabetes and antihypertensive medications [21]. Three years later, Ahmetov et al. reported that the *PPP3R1*:rs3039851 polymorphism was associated with the risk of left ventricular hypertrophy in Russian athletes [22]. However, no current data could be found concerning a possible association between the *PPP3R1*:rs3039851 polymorphism and LVM variance in human subjects not exposed to long-term hypertension or sports training, i.e., environmental factors with a documented ability to activate calcineurin and induce myocardial hypertrophy. Therefore, the aim of this study is to assess possible association between the *PPP3R1*:rs3039851 polymorphism and left ventricular mass measured in full-term healthy newborns.

## 2. Materials and Methods

### 2.1. Newborns

The studied cohort consisted of 162 healthy Polish newborns (71 females and 91 males), born after the end of the 37th week of gestation (from 38 to 40 weeks) to healthy women with uncomplicated pregnancies. Newborns in this study were of appropriate size for their gestational age (defined as birth mass above the 10th centile). Exclusion criteria were the following: twins, intrauterine growth restriction, chromosomal aberrations and/or congenital malformations; congenital malformations of the heart were excluded on the basis of ultrasound images. At birth, newborn cord blood (500 μL) was obtained for isolation of genomic DNA. The sex of the newborn, body mass (BM, kg) and body length (BL, m) were taken from standard hospital records. Body surface area (BSA, m^2^) was calculated using the Mosteller formula [23]:

BSA = 0.0167 × (100 × BL)^0.5^ × BM^0.5^

The study was approved by the Bioethics Committee at the Pomeranian Medical University, Szczecin, Poland; parents gave informed consent, and the study was performed according to the latest Declaration of Helsinki (2013).

### 2.2. Echocardiographic Measurements

Echocardiographic measurements in newborns, on the third day after delivery, were made by one pediatric cardiologist. Two-dimensional M-mode echocardiography was performed (using an Acuson Sequoia 512 unit, Siemens Healthineers, Erlangen, Germany; equipped with a 2–4 MHz imaging transducer). Measurement techniques were consistent with American Society of Echocardiography conventions. Left ventricular masses (LVMs) were estimated from the echocardiographic left ventricular dimension measurements using the Penn convention, with the equation modified by Huwez et al. [24] as follows:

LVM [g] = 1.04 × [(IVST [mm] + LVPWT [mm] + LVID [mm])^3^ − LVID [mm]^3^]

where IVST, LVPWT and LVID denote Interventricular Septal Thickness, Left Ventricular Posterior Wall Thickness and Left Ventricular Internal Dimension, respectively. To give standardized parameters, the left ventricular mass was divided by body mass (LVM/BM, g/kg), body length (LVM/BL, g/m) or body surface area (LVM/BSA, g/m^2^).

### 2.3. PPP3R1 Genotyping

Genomic DNA from cord blood was isolated (using a QIAamp Blood DNA Mini Kit; QIAGEN, Dusseldorf, Germany), according to the manufacturer’s protocol. For the analysis of the *PPP3R1*:rs3039851 (NC_000002.12:g.68218182_68218190: TTAATTTAA, referred to here as the major or 5I allele; NC_000002.12:g.68218186_68218190del: delTTTAA, referred to here as the minor or 5D allele and NC_000002.12:g.68218186_68218190dup: dupTTTAA, a very rare allele) polymorphism, a polymerase chain reaction-restriction fragment length polymorphism (PCR/RFLP) method was applied with the following primer pair: forward: 5′-GAGTTTAAAAGCCAGCCAGTCAT-3′, and reverse: 5′-AAAACAGTAA TATCTCAAGTAGTA-3′ (from TIB MOL BIOL, Poznań, Poland). The *PPP3R1* amplicons were subsequently digested with the VspI (AseI) restriction enzyme (Thermo Fisher Scientific, Waltham, MA, USA). The PCR product was cut into fragments of 201 base pairs (bp) and 103 bp in the presence of the 5I allele or remained uncut in the presence of the 5D allele (with an amplicon of 299 bp in length). Restriction products in each case were electrophoretically separated and visualized by staining (using Midori Green; Nippon Genetics Europe, Duren, Germany) in 3% agarose gels. To verify the results, DNA samples from 16 (10%) randomly chosen newborns were sequenced. Briefly, PCR amplification products were purified using Exonuclease I and FastAP Thermosensitive Alkaline Phosphatase (ThermoFisher Scientific) according to manufacturer procedures. The products were sequenced (using BigDye^®^ Terminator v3.1 Cycle Sequencing Kits; Applied Biosystems, Life Technologies Polska, Warsaw, Poland). Electrophoresis and analyses were performed according to manufacturer procedures (using an ABI PRISM 3100-Avant machine; Data Collection Software v2.0, Sequencing Analysis Software v5.4; Applied Biosystems). In each case, the result obtained by sequencing was as expected from the PCR-RFLP results. All DNA samples were genotyped blind, i.e., the samples were anonymously labeled by one person and then genotyped by a second person.

### 2.4. Statistical Analyses

Quantitative data were tested for normality using Kolmogorov–Smirnov or Lilliefors tests. As most quantitative variables were not normally distributed, all are presented as medians with minimum and maximum values. Quantitative data were compared, e.g., between *PPP3R1* genotype groups (5I/5I vs. 5I/5D + 5D/5D), using Mann–Whitney tests. Categorical data and the divergence of *PPP3R1*:rs3039851 genotype frequencies from Hardy–Weinberg equilibrium were assessed using chi-squared tests. Statistical significance was defined as *p* < 0.05. All data were analyzed using a data analysis software system (Statistica, version 13, TIBCO software, Palo Alto, CA, USA, accessed on 14 November 2022).

## 3. Results

In the sample there were 135 (83.3%) reference (5I/5I) homozygotes, 26 (16.1%) 5I/5D heterozygotes and 1 (0.6%) 5D/5D homozygote. The frequency of the minor *PPP3R1* 5D allele was 8.6%. In the female subgroup (n = 71), there were 56 (78.9%) 5I/5I homozygotes, 14 (19.7%) heterozygotes and 1 (1.4%) 5D/5D homozygote. The frequency of the minor 5D allele in female newborns was 11.3%. In the male subgroup (n = 91), there were 79 (86.8%) 5I/5I homozygotes and 12 (13.2%) 5I/5D heterozygotes, and the frequency of the minor 5D allele was 6.6%. The distributions of *PPP3R1* genotypes or alleles between both subgroups (female newborns versus male newborns) were not significantly different (*p* = 0.178 for genotypes and *p* = 0.137 for alleles), and the *PPP3R1*:rs3039851 genotype distribution was consistent with the Hardy–Weinberg equilibrium in the whole group (*p* = 0.834) as well as in female newborns (*p* = 0.907) and in male newborns (*p* = 0.501).

Characteristics of the newborn cohort with regard to sex are shown in Table 2. There were no significant differences between female newborns and male newborns in the values of body length, left ventricular dimensions (IVST, LVPWT and LVID) or LVM and LVM indices (LVM/BM, LVM/BL and LVM/BSA). Body mass and BSA in male newborns were significantly higher as compared to females.

There were no significant differences with regard to *PPP3R1*:rs3039851 genotype (5I/5I versus 5I/5D + 5D/5D) in the values of clinical variables, left ventricular dimensions or in LVM and LVM indices (Table 3).

There were also no significant differences in the distributions of *PPP3R1* genotypes between newborns with the highest (above median) or with the lowest (below median) values of LVM indices (Table 4). In addition, we compared the *PPP3R1* polymorphism distribution between newborns in the lower tertile (LT) and newborns in the upper tertile (UT) for LVM indices (Table 5). The frequency of *PPP3R1* genotypes with at least one 5D allele (5I/5D + 5D/5D genotypes) was significantly higher for LVM/BM and LVM/BSA in newborns from the upper tertile as compared with lower tertile subjects.

## 4. Discussion

Left ventricular mass is a continuous quantitative trait that is modulated by a complex interaction between genetic and environmental factors [25]. Previously, it has been pointed out that healthy full-term newborns, due to the lack of confounding environmental factors such as diet, lifestyle, smoking, coexisting diseases or medication, are the optimal population group for identifying genetic variants determining (or modulating) cardiovascular phenotypes [26]. Despite this, there are very few reports on this issue in relation to left ventricular mass [25,27,28,29].

Our study reports on the frequency of *PPP3R1*:rs3039851 genotypes in healthy full-term newborn Poles with respect to left ventricular mass. We have studied newborns born in Szczecin who are descendants of people who moved to Polish West Pomerania after World War II from many parts of the former Poland [30,31]. This migration is considered to be the main reason why contemporary inhabitants of this region are considered a representative sample for the entire Polish population [32,33]. Therefore, the frequency of the *PPP3R1* 5D allele determined in newborns from Szczecin, at 8.6%, can be treated as a value likely corresponding to the actual prevalence of this genetic variant among Poles. In addition, a very similar 5D frequency (8.8%) was found by Akhmetov et al. in another group of Slavic people consisting of 1073 young healthy Russians [22]. Tang et al.’s study found that the frequency of the *PPP3R1* 5D allele in people of European descent was 7.6%, and among African Americans as high as 23.9% [21]. Similar differences in the prevalence of the *PPP3R1* 5D allele were also noticed by Hand et al. who, in a group of white people in the USA, determined the frequency of this variant at 7% and in a group of African-Americans (n = 33) at 24% [34]. In addition, the *PPP3R1* 5D allele frequency in Polish newborns is consistent with the results of rs3039851 genotyping in populations of European descent included in the 1000 Genomes (1KG) Project (ensembl.org).

Previous studies have associated the *PPP3R1* 5I/5D polymorphism with left ventricular mass in adults exposed either to the long-term effects of hypertension [21] or to sports training [22], i.e., environmental factors with a documented ability to both activate calcineurin and induce myocardial hypertrophy [9,20,35,36]. The results of our study have shown that the *PPP3R1* 5I/5D polymorphism is also likely to be associated with left ventricular mass in healthy subjects not exposed to these risk factors.

Evidence for a significant role of calcineurin in developmental mechanisms determining normal heart mass was provided in 2002 [14]. Bueno et al., in transgenic mice lacking the gene encoding the beta isoform of the calcineurin catalytic subunit, showed that at eight weeks after birth, the heart mass of these young animals was significantly (12%) lower compared to mice of the same age without a defect in the gene [14].

Tang et al. indicated that the deletion of 5 nucleotides (5D allele) in the *PPP3R1* promoter results in the abolition of a potential binding site of the transcription factor NKX-2, which plays an important role in the regulation of myogenesis and myocardial development [37,38]. In addition, Schott et al. and Benson et al. have shown that mutations in the *NKX2-5* gene cause congenital heart defects in humans [39,40]. Therefore, Tang et al. hypothesized that the NKX-2 motif is an important binding site for a repressor or inhibitor of Calcineurin subunit B type 1 transcription at or near the promoter region, and the deletion of 5 nucleotides in this region leads to increased expression of this gene and, consequently, increased calcineurin activity [21].

**Figure 1:**
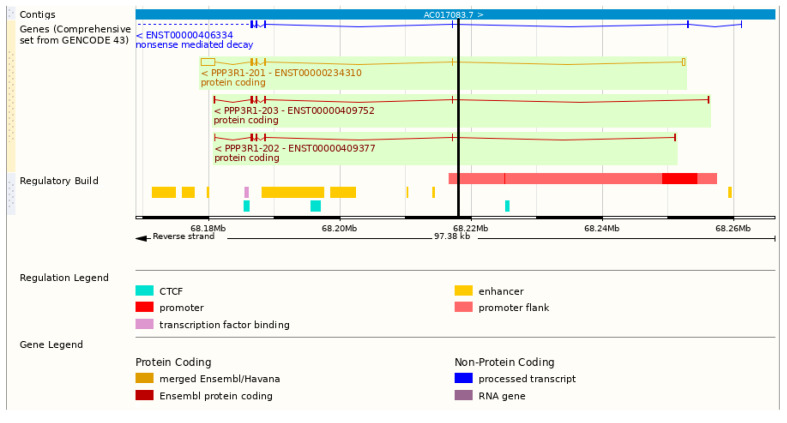
Genetic annotation of *PPP3R1* (for full details, see Ensembl: ENSG00000221823). Showing transcript relationships including a promoter region. The location of the rs3039851 insertion/deletion is shown by the thick vertical black line in the first intron (*PPP3R1* is reverse strand, so this and the promoter are on the right-hand side). Source of image: modified from Ensembl version 109 [41].

However, this hypothesis now apparently conflicts with the evidence from the three Ensembl transcripts (the reference/canonical transcript: ENST00000234310.8 plus ENST00000409752.5 and ENST00000409377.1, referred to here as A, B and C, respectively) (Figure 1).

All of these transcripts are assigned a promoter further upstream giving an additional exon, exon 1, with numbers of amino acids predicted to be translated from this exon: A, 1; B, 20, C, 0. The *PPP3R1*:rs3039851 Indel is therefore assigned as being in Intron 1–2 and not, therefore, at or near a promoter region.

There are various ways in which the above conflict could be resolved, and all include re-evaluation of available transcripts and, possibly, the addition of missing, alternative haplotype transcripts. It is rather unlikely that Transcript A is incorrect as it has been given a high-fidelity transcript tag by Ensembl (tsl1; meaning “all splice junctions of the transcript are supported by at least one non-suspect mRNA”). However, perhaps the first exon does not form part of transcript C (which has tag tsl3: “The only support is from a single EST”)? This would then give transcripts which would conform to the situation predicted by Tang et al. The 5I allele would then have a predicted TATA-box promoter region containing the Indel: the Eukaryotic Neural Network Promoter Prediction tool, NNPP, at https://www.fruitfly.org/seq_tools/promoter.html (accessed 22 February 2023) gave a score of 0.92 for the region: aagatatatatttaaatatgcccatgttaattgtacacttAaattaatag (Indel and duplication underlined) and a higher score of 0.95 for the 5D allele: aagatatatatttaaatatgcccatgtt aattgtacacttAatagcaact, which might also indicate, together with the lack of an NKX-2 binding site, a propensity for higher expression, as suggested by Tang et al.

Re-evaluation of transcripts, collection of new transcripts and prevalence of the three different *PPP3R1* polypeptides with lengths: A, 170; B, 189 and C, 160 amino acids, produced by the splice variants, are now urgently needed. Further, we are not able to rule out whether the Indel affects transcription from an intronic location, or, as previously stated by Tang et al. [11], whether the *PPP3R1*:rs3039851 polymorphism is only in close linkage disequilibrium with some, as yet unidentified, variant of real functional importance. This hypothesis seems to be strongly supported by Alsheikh et al., who indicated an urgent need to assess the actual functional significance of genetic polymorphisms associated with predisposition to specific diseases identified in genome-wide association studies, of which as many as 90% are non-coding variants [42].

We are fully aware that a major limitation of our study is its relatively low statistical power. This results mainly from the low prevalence of the *PPP3R1*:rs3039851 polymorphism in studied newborns (8.6%) and from a relatively small sample size (n = 162). Data from the 1KG Project (https://www.internationalgenome.org/) clearly confirm that the frequency of the *PPP3R1* 5D variant, at <10%, is typical for populations of European descent and is about three times lower than in subjects of relatively recent African descent. In addition, by using the Open Epi (www.openepi.com) tool, a free and open source software for epidemiologic statistics, we have computed the minimum sample size for 80% statistical power and 5% type I error rate: assuming a ratio of *PPP3R1* 5I/5I homozygotes to carriers of at least one 5D allele (5D+) equal to 5.0 (135/27) and LVM/BL higher than the median in 15 of 27 (56%) 5D+ newborns and in 66 of 135 (49%) 5I/5I newborns. Under the above assumptions, the minimum sample size necessary varied from 3113 to 3220 (519–537 of 5D+ and 2594–2683 of 5I/5I newborns). On the other hand, Tang et al. showed a significant association between the *PPP3R1* polymorphism with inappropriately high LVM by studying 197 African-American patients only, but 35% of these were carriers of at least one 5D allele [21].

## 5. Conclusions

Our results suggest that the *PPP3R1*:rs3039851 polymorphism may account for subtle variation in left ventricular mass in full-term healthy newborns.

## Figures and Tables

**Table 1:** Calcineurin-subunit-dependent cardiovascular phenotypes, based on mouse homologs.

Calcineurin Subunit Abbreviation (Recommended Name: Human) UNIPROT References: Human/Mouse	Human Gene (*locus*) Mouse Homolog	Cardiovascular Phenotypes [Reference]
CnAα (Protein phosphatase 3 catalytic subunit alpha) Q08209/P63328	*PPP3CA* (4: 101,023,409–101,348,278) *Ppp3ca*	Association of rs3017892, rs1441433, rs12116679 and rs1527351 with blood pressure [10,11,12]. Cardiac hypertrophy and development of heart failure within the first few weeks of age in transgenic mice overexpressing a constitutively active form of CnAα [13].
CnAβ (Serine/threonine-protein phosphatase 2B catalytic subunit beta isoform) P16298/P48453	*PPP3CB* (10: 73,436,433–73,496,024) *Ppp3cb*	Association of 12,644 with systolic blood pressure [11]. A 80% decrease in calcineurin enzymatic activity associated with a 12% reduction in basal heart size in CnAβ-deficient mice [14]. No induction of cardiac hypertrophy but improvement of cardiac function and reduction of scar formation after myocardial infarction in transgenic mice with overexpression of CnAβ1 isoform (splicing variant of CnAβ gene with unique C-terminal region) in postnatal cardiomyocytes [15].
CnAγ (Serine/threonine-protein phosphatase 2B catalytic subunit gamma isoform) P48454/P48455	*PPP3CC* (8: 22,440,819–22,541,142) *Ppp3cc*	Association of 73,227,871 with SBP [16].
CnB1 (Calcineurin subunit B type 1) P63098/Q63810	*PPP3R1* (2: 68,178,857–68,256,237) *Ppp3r1*	Association of rs6731373 with SBP and with pulse pressure [17]. Lethality at 1 day after birth due to altered right ventricular morphogenesis, reduced ventricular trabeculation, septal defects and valvular overgrowth in mice with the cardiomyocyte-specific deletion of CnB1 in embryonic development. Lethality in early mid adulthood with reduction in cardiac contractility, severe arrhythmia and reduced cardiomyocyte content in mice with postnatal-deletion of CnB1 [18]. Lethal cardiomyopathy, partially characterized by impairment of postnatal growth of cardiac-specific CnB1-deficient mice [19].
CnB2 (Protein phosphatase 3 regulatory subunit B, beta)	*PPP3R2* (9: 101,591,604–101,595,021) *Ppp3r2*	No data.

**Table 2:** Clinical and echocardiographic characteristics of the newborns with regard to sex.

Variable	Female Newborns (n = 71)	Male Newborns (n = 91)	*p*
	Median (Minimum–Maximum)		
BM [kg]	3.32 (2.64–4.30)	3.56 (2.65–5.09)	0.014
BL [m]	0.55 (0.51–0.63)	0.56 (0.47–0.63)	0.311
BSA [m^2^]	0.230 (0.195–0.270)	0.234 (0.199–0.289)	0.037
IVST [mm]	3.9 (2.4–6.3)	3.7 (2.4–5.1)	0.139
LVPWT [mm]	2.7 (1.1–5.8)	2.8 (2.0–4.0)	0.307
LVID [mm]	18.3 (16.0–25.0)	18.1 (15.0–23.2)	0.402
LVM [g]	9.4 (4.9–21.9)	9.7 (5.4–17.6)	0.552
LVM/BM [g/kg]	2.8 (1.5–6.4)	2.9 (1.8–5.6)	0.962
LVM/BL [g/m]	16.8 (9.0–35.9)	17.8 (10.0–32.1)	0.790
LVM/BSA [g/m^2^]	53.3 (29.0–116.0)	55.9 (34.5–106.3)	0.869

Legend: BM = Body Mass; BL = Body Length; BSA = Body Surface Area; IVST = Interventricular Septal Thickness; LVPWT = Left Ventricular Posterior Wall Thickness; LVID = Left Ventricular Internal Dimension, LVM = Left Ventricular Mass. *p* values are from Mann–Whitney tests.

**Table 3:** Clinical and echocardiographic characteristics of the newborns in regard to *PPP3R1* genotype (5I/5I or 5I/5D + 5D/5D).

Variable	5I/5I (n = 135)	5I/5D + 5D/5D (n = 26 + 1)	*p*
	Median, Minimum–Maximum		
BM [kg]	3.37, 2.64–4.48	3.47, 2.84–5.09	0.432
BL [m]	0.56, 0.47–0.63	0.56, 0.50–0.60	0.572
BSA [m^2^]	0.230, 0.195–0.276	0.232, 0.203–0.289	0.643
IVST [mm]	3.8, 2.4–6.1	3.8, 2.8–6.3	0.955
LVPWT [mm]	2.8, 1.1–5.8	3.0, 1.6–5.0	0.317
LVID [mm]	18.4, 15.0–25.0	18.1, 15.0–22.0	0.579
LVM [g]	9.7, 4.9–21.9	9.4, 6.0–19.5	0.946
LVM/BM [g/kg]	2.8, 1.5–6.4	3.0, 1.9–5.2	0.590
LVM/BL [g/m]	17.4, 9.0–35.9	18.3, 11.0–33.3	0.794
LVM/BSA [g/m^2^]	42.0, 22.3–90.6	44.7, 27.9–78.8	0.650

Legend: For abbreviations see Table 1. *p* values are from Mann–Whitney tests.

**Table 4:** Frequency distributions of *PPP3R1* genotypes (5I/5I or 5I/5D + 5D/5D) in newborns with regard to left ventricular mass indices.

Variable	Median	Group	5I/5I n (%)	5I/5D + 5D/5D n (%)	*p*
		<2.8	68 (83.9)	13 (16.1)	
LVM/BM [g/kg]	2.8				0.833
		>2.8	67 (82.7)	14 (17.3)	
		<17.5	69 (85.2)	12 (14.8)	
LVM/BL [g/m]	17.5				0.527
		>17.5	66 (81.5)	15 (18.5)	
		<42.2	68 (83.9)	13 (16.1)	
LVM/BSA [g/m^2^]	42.2				0.833
		>42.2	67 (82.7)	14 (17.3)	

Legend: For abbreviations see Table 1. *p* values are from chi-squared tests.

**Table 5:** Frequency distributions of *PPP3R1* genotypes (5I/5I or 5I/5D + 5D/5D) in newborns from lower or upper tertiles of left ventricular mass indices.

Variable	Tertile	5I/5I n (%)	5I/5D + 5D/5D n (%)	*p*
	Lower: ≤2.6	49 (90.7)	5 (9.3)	
LVM/BM [g/kg]				0.007
	Upper: ≥3.1	38 (70.4)	16 (29.6)	
	Lower: ≤15.5	47 (87.0)	7 (13.0)	
LVM/BL [g/m]				0.056
	Upper: ≥19.4	39 (72.2)	15 (27.8)	
	Lower: ≤38.4	48 (88.9)	6 (11.1)	
LVM/BSA [g/m^2^]				0.029
	Upper: ≥45.8	39 (72.2)	15 (27.8)	

Legend: For abbreviations see Table 1. *p* values are from chi-squared tests.

## Data Availability

The data are available from the corresponding author upon reasonable request.
